# Information management of full-cycle inpatient bed reservation for cancer patients under the normalised prevention and control of the COVID-19 pandemic

**DOI:** 10.1186/s12913-024-11206-6

**Published:** 2024-07-12

**Authors:** Yang Yang, Yang Bin, Ma Yanping, Zhao Jinping, Zhou Xin, Cheng Chunjun, Zhai Zhenhua

**Affiliations:** 1 Department of Breast, Head and Neck Oncology, First Affiliated Hospital of Jinzhou Medical University, No. 2 Duan 5, Renmin Street, Guta District, Jinzhou, 121001 Liaoning Province China; 2 Tumor Vascular and Microenvironment Laboratory, First Affiliated Hospital of Jinzhou Medical University, Jinzhou, China; 3 College of International Education, Jinzhou Medical University, Jinzhou, China

**Keywords:** Normalisation of pandemic prevention and control, Cancer patients, Bed reservations, Operational efficiency, Nursing management

## Abstract

**Background:**

During the prolonged COVID-19 pandemic, hospitals became focal points for normalised prevention and control. In this study, we investigated the feasibility of an inpatient bed reservation system for cancer patients that was developed in the department?s public WeChat account. We also explored its role in improving operational efficiency and nursing quality management, as well as in optimising nursing workforce deployment.

**Methods:**

We utilised WeChat to facilitate communication between cancer patients and health care professionals. Furthermore, we collected data on admissions, discharges, average number of hospitalisation days, bed utilisation rate, and the number of bed days occupied by hospitalised patients through the hospital information system and nurses? working hours and competency levels through the nurse scheduling system. The average nursing hours per patient per day were calculated. Through the inpatient bed reservation system, the number of accepted admissions, denied admissions, and cancelled admissions from the reservation system were collected. The impact of the bed reservation system on the department?s operational efficiency was analysed by comparing the number of hospitalisation discharges before and after reservations, as well as the average hospitalisation and bed utilisation rates. By comparing nurses? working hours per month and average nursing hours per patient per day, the system?s impact on nurses? working hours and nursing quality indicators was analysed.

**Results:**

The average hospitalisation length, bed utilisation rate, and nurses? working hours were significantly lower, and the average number of nursing hours per patient per day was significantly higher after the implementation of the reservation system. The full-cycle bed information management model for cancer patients did not affect the number of discharged patients.

**Conclusion:**

Patients? ability to reserve bed types from home in advance using the department?s official WeChat-based inpatient bed reservation system allowed nurses to prepare for their work ahead of time. This in turn improved the operational efficiency of the department and nursing quality, and it optimised the deployment of the nursing workforce

## Background

The International Agency for Research on Cancer of the World Health Organization released the latest global cancer burden data in 2020, which showed that China has the highest number of new cancer cases and cancer-related deaths globally [1]. Patients with cancer are highly dependent on inpatient services and require hospitalization during a third of the entire treatment cycle [2]. Bed resources constitute an essential component of medical resources in hospitals, and the reasonable allocation and utilisation of bed resources can reflect the productivity of departments and hospital management [3]. The COVID-19 pandemic has spread globally since the beginning of 2020. Confronted with such an acute public health event, infection risk mitigation among medical staff and nurses is necessary, requiring pandemic changes and prevention and control measures. Simultaneously, in the face of significant operational difficulties faced by hospitals, improving the continuous efficiency of high-quality medical services is imperative to reduce the risk of patients transmitting infection between hospitals and communities [4] and ensure that multiple treatment needs and the safety of cancer patients are met across the full treatment cycle. Through inpatient bed reservations on the internet+, cancer patients can fully experience efficient, fast, and comfortable medical services with welcoming human staff [5].

In China, WeChat, a free social application accessible on both smartphones and web browsers, is immensely popular among people of all ages. Through WeChat, users can effortlessly exchange messages; communicate via multimedia options, including text, voice, and video; explore topics of interest; and engage with public WeChat accounts [6]. WeChat is easy to operate and offers multiple functions. It has been reported to be an effective tool for the management of chronic diseases such as cancer [7]. Moreover, it has been reported that hospitals’ public WeChat accounts can promote people’s health and equity in accessing medical information and services [8].

During the prolonged COVID-19 pandemic, hospitals were focal points for normalised prevention and control. Due to the chronic shortage of inpatient beds in China’s large hospitals during this time, patients faced difficulties in securing admission, resulting in prolonged waiting times for beds. It is worth considering shifting from a scenario of ‘people waiting for beds’ to one where ‘beds await people’, aiming to alleviate the plight of patients denied admission due to bed shortages, mitigate the risk of COVID-19 transmission within the hospital and community, and manage the surge in patients efficiently. Implementing online bed reservation reviews before admission, rather than in person, has emerged as a convenient and efficient measure for reducing hospital crowding and curbing the spread of COVID-19.

Since WeChat is already widely used in China, as previously described, and has been proven to be effective in improving medical services, this study aimed to investigate the feasibility of using an inpatient bed reservation system for cancer patients developed in the department’s public WeChat account and its potential role in improving the department’s operational efficiency, nursing workforce management, and nursing quality under the normalised prevention and control of the COVID-19 pandemic.

## Methods

The First Affiliated Hospital of Jinzhou Medical University was selected for this study. It is a large tertiary general hospital in western Liaoning. The medical wards of the breast, head, and neck oncology departments were the first to introduce the full-cycle management of beds for cancer patients and served as the research object. The ward has 35 open beds. During the study period, no significant differences were detected in nurses’ competency levels in the wards before and after implementing the bed reservation system (*P* = 0.126) (see Table 1). Nurses’ competency levels were scored by the ‘Nurses competency levels assessment scoring standard’ and divided into five levels (N0–N4) according to nurses’ work ability, title, years of work experience, and education level. The higher the nurses’ scores were, the greater the nurses’ professional competency. This area had a low COVID-19 risk. All patients admitted were from low- and medium-risk areas. All hospitalised patients fulfilled the requirements for hospitalisation; after undergoing epidemiological investigations and nucleic acid tests, none were suspected or confirmed to have COVID-19.

**Table 1:** Competency levels of ward nurses before and after using the bed reservation system

		Before bed reservation (*n* = 114)	After bed reservation (*n* = 111)
Competency Levels of Nurses	N0	22(19.3%)	14(12.61%)
	N1	37(32.46%)	52(46.85%)
	N2	37(32.46%)	33(29.73%)
	N3	18(15.79%)	12(10.81%)
χ^2^	5.73		
P	0.126*		

*P > 0.05 indicates that the difference was not statistically significant

### Bed reservation system design

The public WeChat bed reservation information management platform, with department-specific characteristics, was developed in collaboration with Shanghai Ruochu Information Technology Co., Ltd. The platform consists of the physician’s terminal and the patient’s terminal. The office nurse in charge of bed approvals and the head nurse were responsible for WeChat communication through the doctor’s terminal. In contrast, the patient’s terminal was integrated into the department’s public WeChat account platform, rendering it convenient for patients to access and use.

1. Before-bed reservation

The conventional inpatient model was adopted between February and December 2020, and beds were not reserved. When patients visited the outpatient clinic, they were admitted to the hospital on the same day if there were vacant beds in the ward. If the wards were full, the patients would leave their contact details and wait at home.

2. After-bed reservation.

A bed reservation information management model was adopted between February and December 2021. Patients used the inpatient bed reservation system developed in a public WeChat account to make reservations for inpatient beds three days in advance. If the supply of reserved beds or individual demand for beds was unmet, the nursing team dynamically adjusted the beds, communicated with patients, and attempted to satisfy their demand as much as possible. If the patient’s needs were unmet, they could make reservations for a bed later.

**Fig. 1:**
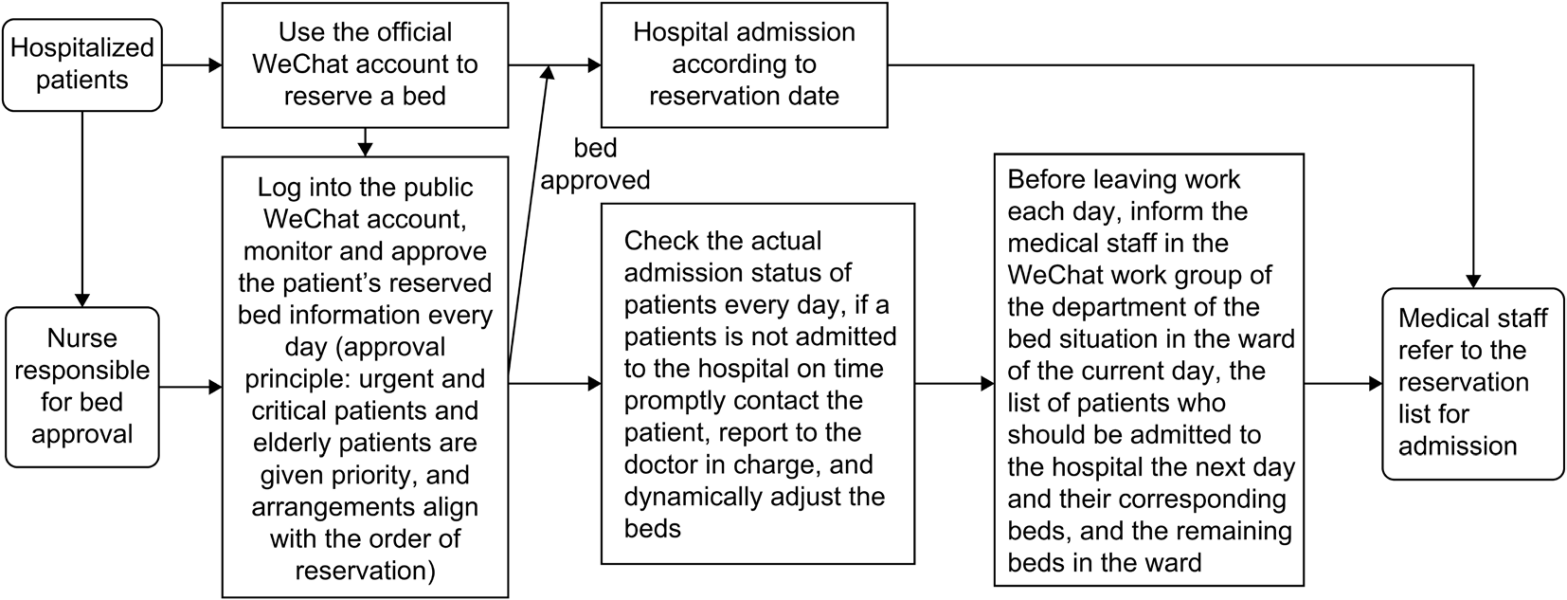
Bed reservation system and its standardised process

3. The bed reservation system and its standardised process (Fig. 1).

Admitted patients used the public WeChat account for inpatient bed reservations. The bed number was reserved three days in advance. The reservation of the number of resources for the next day closed at 16:30 each day. After the patients received approval for bed allocation, they were admitted to the hospital based on the reservation date. The nurse checked the bed reservation situation daily and approved the beds by 16:30 the day before the patient’s scheduled admission date. The principles of bed arrangement prioritize urgent and critical, with older adult patients being given priority and arrangements aligning with the reservation order. The nurses were responsible for approving the reserved beds daily. The actual admission status of the patients was monitored daily. If a patient was not admitted on time, the nurse contacted the patient promptly, reported to the doctor in charge [9], and dynamically adjusted the bed allocation. Before leaving work every day, the medical staff was informed of the bed situation in the wards for that day, the list of patients who should be admitted to the hospital the next day, their corresponding bed numbers, and the remaining beds in the ward (with a list to refer to for admissions).

4. Application of the bed reservation information management platform.

4.1 Patient’s terminal of the bed reservation platform

**Fig. 2:**
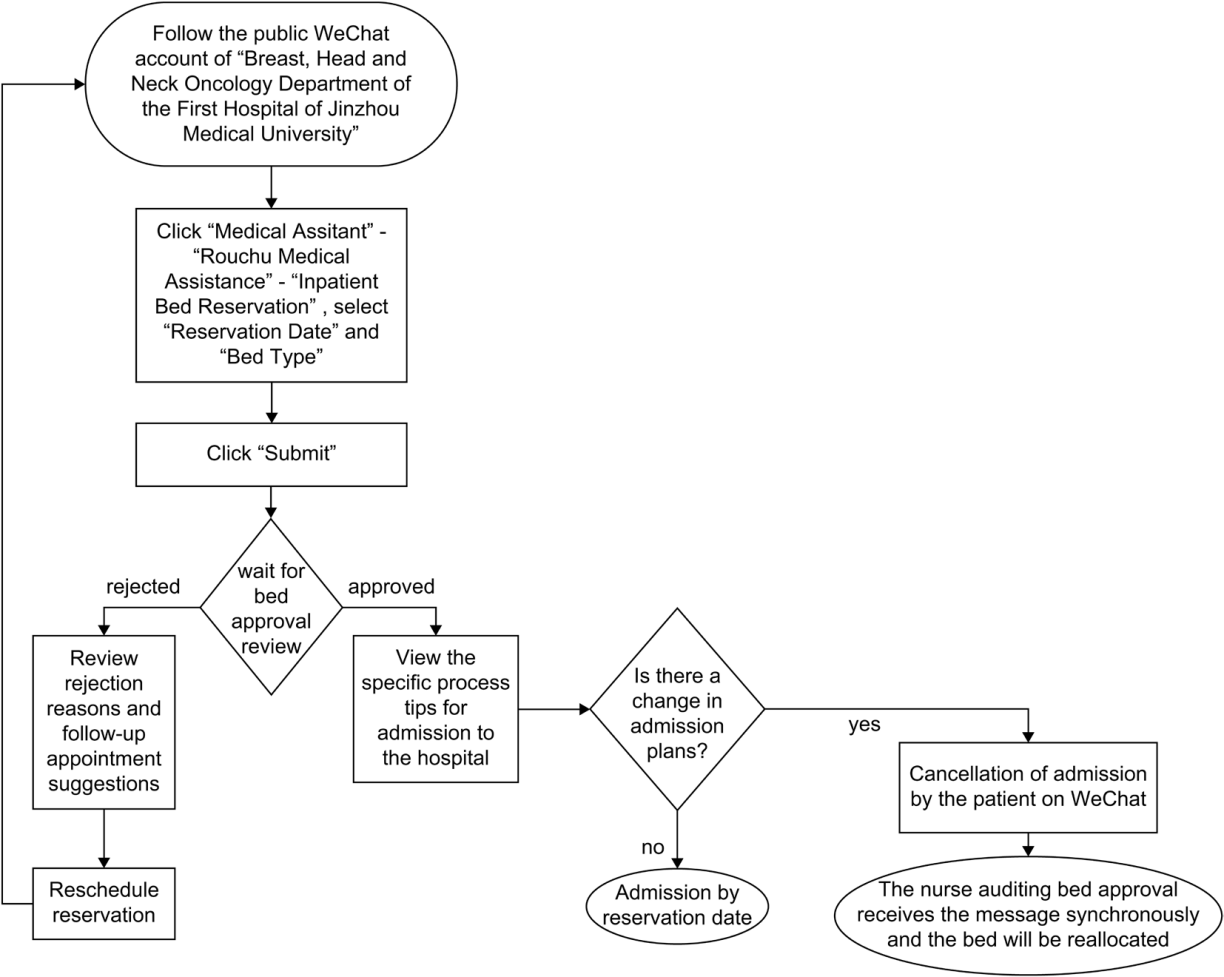
Method of using the patient’s terminal

Figure 2 shows a schematic of how to use the patient terminal. First, to make a reservation, the patient followed the official WeChat account of the Department of Breast, Head and Neck Oncology, First Hospital of Jinzhou Medical University, clicked ‘Medical Assistant’→ ‘Ruochu Medical Assistance’ → ‘Inpatient Bed Reservation’, selected ‘Reservation Date’ and ‘Bed Type’, clicked the ‘Submit’ button and waited for review. Second, in the message reception phase, after reviewing the bed, the patient’s WeChat automatically received a new push message from the department’s official WeChat account to inform them if the bed reservation was approved. If the reservation was approved, the patient was reminded of the specific procedures for admission. Otherwise, the reasons for rejection and suggestions for follow-up reservations were provided. The third step was cancelling the reservation. If the admission needed to be cancelled, the patient could cancel it on WeChat, and the nurse’s review terminal simultaneously received the notification of bed cancellation.

4.2 Physician’s terminal of the bed reservation platform

The head nurse and the office nurse logged into their personal WeChat account, entered the personal QR code of the ‘Ruochu Medical Assistant Professional Edition’, and checked the reservation status of the inpatient beds. Bed approval was obtained daily, including the name, age, and contact number of the patient who made the reservation, reservation date, and reserved bed type. The approval result was either ‘Approved’ or ‘Rejected’. When a bed reservation was refused, the nurse described the reason and made suggestions for follow-up reservations in the column for reasons of refusal. The overall summary of patients who made reservations was made available in the ‘Pending Review’, ‘Approval’, and ‘Audit Record’ modules.

### Evaluation indicators

The indicators used to measure the operational efficiency of the departments were the number of discharges, average hospitalisation days, and bed occupancy rate. Nurse working hours were measured per month, which reflects the hospital’s workforce management. The control indicator of nursing quality was the average number of nursing hours per patient per day.

In 2020, the National Health Commission of the PRC released the ‘Medical Quality Control Indicators for Nursing Professionals (2020 Edition)’, which marked the first instance where nursing indicators were integrated into monitoring medical quality control at the national level [10]. The average number of daily nursing hours per patient has gained attention as a quality control indicator. This indicator refers to the ratio of the actual number of nurses working in medical institution wards to the actual number of inpatient bed days. It reflects the average daily nursing hours that each hospitalised patient receives. Monitoring it can guide managers in allocating nursing staff appropriately, promoting nursing productivity, and allowing nurses to devote more of their working time to patient care.

### Data collection and analysis

The hospital information system (HIS) documented every patient hospitalisation. Through the HIS, our department collected data on admissions, discharges, average number of hospitalisation days, bed utilisation rate, and number of bed days actually occupied by hospitalised patients. Through the nurse scheduling system, nurses’ working hours and competency levels were collected. The average nursing hours per patient per day were calculated by dividing the number of hours actually worked by licensed nurses in the medical institution’s wards by the number of bed days actually occupied by inpatients. The inpatient bed reservation system on the department’s public WeChat account recorded information on patient bed reservations. The number of accepted admissions, denied admissions, and cancelled admissions were collected using the inpatient bed reservation system. To evaluate departmental operational efficiency, the number of discharges, average number of hospitalisation days, and bed utilisation rate before and after bed reservation were compared. The number of hours of nurses’ work per month was compared to analyse the impact of bed reservations on nurses’ working hours. By comparing the average number of nursing hours per patient daily, we analysed the impact of the implementation of bed reservations on the nursing quality indicators.

### Statistical methods

SAS-jmp11.0 (SAS Institute, Inc.) statistical software was used for data analysis, with *P* < 0.05 considered to indicate a statistically significant difference. The competency level of nurses in the ward was considered a categorical variable, and the chi-square test was used for analysis. The number of discharges, average hospitalisation days, bed occupancy rate, working hours of nurses, and average nursing hours per patient per day were numerical variables, and the Kolmogorov‒Smirnov test was used to determine whether the data were distributed normally. If the data were normally distributed, they were analysed using *t* tests. If the distribution was skewed, the Kruskal–Wallis test by rank was used.

## Results

Hospitalisations increased with an over 95% admission rate after-bed reservation.

The inpatient bed reservation system developed on the public WeChat account has been active since February 3, 2021. Furthermore, 2,143 people have been admitted to the hospital through the bed reservation system since the implementation of the full-cycle bed information management model for cancer patients. Three hundred and six people were refused admission because no beds were available or because the bed type (rooms with one, three, or six beds) did not meet their needs. One hundred and eighty-four people cancelled their appointments because of adjustments in treatment plans that resulted in hospitalisation no longer being needed, being postponed, or encountering last-minute changes. Between February 3 and December 31, 2021, the number of admissions to the ward was 2,228, while the ratio of admissions via reservations to actual admissions was 96.2%. After the bed reservation system was implemented, the total number of admissions was 2,268 from February to December 2021, compared to 2,154 during the same period in 2020 before the reservation system was implemented. The average number of hospitalisations increased by 11 (on average) per month, but no significant difference was found between the groups (*P* = 0.222) (Table 2).

**Table 2:** Number of admissions before and after the use of the bed reservation system

	Before bed reservation (*n* = 11)	After bed reservation (*n* = 11)
Number of admissions	195.82 $\pm$12.50	206.18$\pm$23.94
*t* value	-1.27	
*P* value	0.222	

*P < 0.05

### Improvement in department operational efficiency

In 2020, at the beginning of the pandemic and prior to the bed reservation system being implemented, the total number of discharges throughout the year was 2055, with an average of 186.82$\pm$19.45 per month. In 2021, after the bed reservation system was implemented under the normalised prevention and control of the COVID-19 pandemic, the total number of discharges throughout the year was 2,233, with an average of 203.00$\pm$20.4 per month. No statistically significant difference was found in the number of discharges before and after the implementation of the reservation system (*P* = 0.071). However, after the bed reservation system was implemented, the average number of discharges per month in the department increased by 17. Table 3 shows that the average number of hospitalisation days decreased by 0.99 days (*P* = 0.002), the bed utilisation rate decreased (*P* = 0.023), and operational efficiency significantly improved.

**Table 3:** Comparison of the number of discharges, average length of hospitalisation, and bed utilisation rate before and after the use of the bed reservation system

	Before bed reservation (*n* = 11)	After bed reservation (*n* = 11)		
Number of discharges	186.82$\pm$19.45	203.00$\pm$20.4	t = -1.90	*P* = 0.071
Average number of hospitalisation days	5.45(0.56)	4.46(0.62)	χ^2^ = 9.32	*P* = 0.002*
Bed utilisation rate	0.96$\pm$0.05	0.87$\pm$0.11	t = 2.54	*P* = 0.023*

*P < 0.05

Effects of implementing the bed reservation system.

After implementing the bed reservation system, the median number of working hours of nurses per month decreased from 184 to 176 h (*P* = 0.021) (Table 4). The average number of nursing hours per patient per day increased from 1.73$\pm$0.15 h/day to 1.90$\pm$0.18 h/day (*P* = 0.028) (Table 5).

**Table 4:** Comparison of nurses’ working hours before and after using the bed reservation system

	Before bed reservation (*n* = 114)	After bed reservation (*n* = 111)
Number of nurses’ working hours	184(21)	176(16)
χ^2^	5.31	
P	0.021*	

*P < 0.05

**Table 5:** Comparison of the average number of nursing hours per patient per day before and after the use of the bed reservation system

	Before bed reservation (*n* = 11)	After bed reservation (*n* = 11)
Average nursing hours per patient per day	1.73$\pm$0.15	1.90$\pm$0.18
t value	-2.38	
*P* value	0.028*	

*P < 0.05

## Discussion

The number of inpatient beds in large tertiary hospitals in China is insufficient. This deficiency leads to difficulties with admission, long waiting times, and cumbersome admission procedures. These issues affect the treatment of patients’ diseases and their confidence in overcoming them [11]. When the demand for beds exceeds the hospital’s capacity, health care quality is severely reduced, leading to a worse prognosis for all patients requiring hospitalisation [12]. The waiting time for inpatients is an essential indicator of a hospital’s productivity, medical quality, and management.

In the fight against the COVID-19 pandemic, China’s public hospitals have been the main force, shouldering the most urgent, dangerous, and arduous medical treatment. In addition to meeting the treatment needs of patients and providing high-quality diagnostic and treatment services, multiple measures must be used to prevent and control the pandemic and reduce unnecessary personnel trips and gatherings to minimise the risk of infection in hospitals.

Cancer patients depend heavily on inpatient services and often require multiple long-term treatments throughout each treatment cycle. Under the normalised prevention and control of the COVID-19 pandemic and the lack of new beds in wards, the efficient and rational use of beds to speed up bed turnover is essential to meet cancer patients’ demand for inpatient beds and reduce their waiting time. Guided by patient needs and considering their difficulties and pain points during the process of medical treatment, the nursing service model must be reshaped to expand the scope of nursing services, improve their efficiency, and provide high-quality, accessible, efficient, and empathetic nursing services [13], which will guide nursing managers to continuously explore new ideas.

The rapid development of information technology in the era of big data has promoted the transformation of the modern nursing management model from a traditional paper-based, inefficient model to an information-based, intelligent model [14]. With the in-depth medical reform and implementation of the *Healthy China* strategy, new demands have been raised for public hospitals to improve reservation, diagnosis, and treatment services [15]. Inpatient bed management is critical in hospital patient care [16]. Under the normalised prevention and control of the COVID-19 pandemic, arranging the total patient population and managing the continuously increasing number of patients are also crucial for effective bed management.

However, nursing informatisation is a complex system-engineering process that remains in the preliminary exploratory stage in China [14]. The local and international literature on hospital bed information management is limited. Thus, combining hospital bed management perfectly with modern and convenient information remains difficult [17].

### Characteristics of the inpatient bed reservation system

The inpatient bed reservation system developed in the public WeChat account was used to achieve a full-cycle bed information management model for cancer patients and leverage the advantages of the internet+. Operating the reservation system is simple and easy; it is not limited by space or time. Patients exhibit high compliance levels, reducing unnecessary round trips between the hospital and community and facilitating medical treatment for patients under normalised prevention and control of the pandemic.

This study revealed that between February and December 2021, 2,143 people were admitted to the hospital through the department’s bed reservation system. Three hundred and six patients were rejected from admission, and 184 patients cancelled their reservations, for an admission rate of 96.2%. In other words, after bed reservations were made, 2,143 patients were admitted to the hospital on the planned date and assigned to their preferred beds. This strategy prevented unnecessary round trips between the community and the hospital for 306 patients. One hundred eighty-four beds were promptly allocated to other patients waiting to be admitted after the patient cancelled their reservation. Adopting a bed reservation information platform reduces the no-show rate for reserved beds, with high patient compliance, reducing the number of round trips for patients and the risk of crowd gathering and cross-infection in the hospital, as well as realising the scientific and intelligent management of the admission process [18]. It allows patients to travel less, improves the dissemination of information, and fully leverages the advantages of the internet+, rendering it convenient for patients to seek medical treatment under the normalised prevention and control of the pandemic.

### Improvement in the department’s productivity

The full-cycle bed information management model for cancer patients did not affect the number of discharged patients, but it did shorten the average number of hospitalisation days and appropriately reduced the bed utilisation rate, significantly improving productivity, reducing hidden risks, and bolstering the ability of departments to treat more patients.

This study’s results show that information-based inpatient bed reservations do not affect the number of discharges and can assuage concerns that the bed reservation system causes patient loss.

An information-based bed reservation management model based on official WeChat accounts can shorten the average number of hospitalisation days and significantly improve productivity. Using such a model, medical staff can predict admissions in advance, allowing them to make the appropriate arrangements. Therefore, doctors can efficiently plan patient examinations and treatments in advance, while nurses can appropriately plan the admission of patients and arrange beds in an orderly manner. Office nurses can log onto WeChat to approve beds without manually registering the patient’s reservation information or calling the patient to notify them about hospitalisation. Furthermore, the need to answer frequent calls from patients enquiring about beds is eliminated. Patients must reserve a bed in advance based on their hospitalisation date and follow the WeChat notification of the reservation system to be admitted to the hospital on time. This system saves time for medical staff and patients, assuages patients’ concerns regarding hospital admission, and improves the utilisation efficiency of medical resources [19]. Patients are admitted as scheduled, and the process of admission is streamlined. Ward crowdedness is mitigated during peak hours, and orderly work is ensured. Consequently, limited bed resources can be fully and appropriately utilised. A relatively constant workload and good-quality medical care are guaranteed [9].

Using a public WeChat account to reserve inpatient beds did not affect the number of patients discharged and significantly shortened the average hospitalisation duration. The bed occupancy rate decreased from 96% before implementing the reservation system to 87% after implementation. This difference falls within the reasonable range of the reference index of the bed occupancy rate of tertiary hospitals at 85–93% [20]. Frequent errors caused by excessively high bed utilisation rates, hospital infection control difficulties, heavier medical staff workloads, and uneven medical resource distributions are all reduced, as are the hidden risks associated with these conditions. An increase in medical and nursing work productivity improves the ability of the department to treat more patients while ensuring quality.

### Shortened working hours and high-quality nursing services

The information-based bed management model on the official WeChat account shortened the working hours of nurses and increased the average nursing hours per patient per day, which is beneficial for offering high-quality nursing services, ensuring nursing safety, and providing nurses with a more reasonable amount of rest.

Nurses are an essential part of the medical service system, being the group closest to patients and having the longest interaction time with them. As the foundation of nursing care development, they are indispensable in promoting a healthy China. As of 2018, the number of registered nurses in China was 4.12 million, 2.94 for every thousand persons, far below the 4.45 proposed by the World Health Organization. The general shortage of human resources in nursing poses a potential threat to nursing quality and safety [21].

During the prevention and control period of the COVID-19 pandemic, medical staff in general wards faced the dual pressures of disease treatment and pandemic prevention and control. Meeting the frontline needs of a hospital’s counter-pandemic response while considering personnel responsible for patient care and safety in the department [22] is crucial. This situation has engendered new challenges in managing nursing personnel.

As patient safety is a global issue, the quality of care and safety of patients with cancer have attracted increasing attention, and the timeliness and effectiveness of nursing are essential factors affecting patient safety [23]. Missed nursing care (MNC) refers to the absence or delay of nursing behaviours and functions as an important indicator of nursing quality. The use of MNC has become increasingly mainstream worldwide [24]. Palese et al. (2019) reported that unexpected increases in patient numbers or critical situations, insufficient medical staff, and high admissions and discharges were among the most prominent causes of MNC in Italian health care settings [25]. Min et al. (2020) suggested that ensuring sufficient rest time and a sufficient number of nurses can reduce the occurrence of MNC [26]. Liu et al. (2019) also suggested that insufficient nursing human resources and heavy workloads were the most crucial factors affecting the frequent MNC of oncology nurses [27].

Based on the official WeChat reservation account, the bed information management model assists head nurses in dynamically and flexibly arranging shifts based on total patient numbers in the department, disease classification, admission status, and nursing workload. This system avoids excessive or insufficient workloads by considering nursing work quality and appropriate rest. With an orderly admission process, ward crowds during peak hours can be effectively alleviated, and ward work can be performed in an orderly manner to avoid unexpected increases in the number of patients and a large number of admissions and discharges, resulting in a relatively constant workload. Nurses can enjoy more appropriate rest by shortening their working hours. Increasing the average number of daily nursing hours per patient facilitates high-quality nursing services, nursing safety, and the proper time for nurses to engage with patients more effectively.

### Main discoveries and advantages

It has been reported that bed management improves work efficiency in hospitals. The present study further indicates that reserving inpatient beds prior to admission not only reduces average hospitalisation days but also improves nurses’ efficiency, increasing daily nursing hours per patient. Currently, a few advanced hospitals have centralised admission preparation centres. Patients can apply for admission appointments by entering their personal information into the HIS system and await further contact from staff for admission procedures. Another common method is for patients to contact their doctors or ward nurses’ stations, after which the health care staff manually records requests and notifies the patients by phone or messages when beds become available. However, both methods are inconvenient for patients.

In China, because WeChat is free, easy to use, and has multiple functions, it is popular among people of all ages. The use of WeChat in the management of chronic diseases has been investigated, and improved patient compliance has been reported due to the easy-to-use operation interface and multiple functions of WeChat [28–30]. This study focused on the application of WeChat in inpatient bed reservations, and the results indicated that a bed reservation function based on the WeChat system for cancer patients developed in the department’s public WeChat account was advantageous. First, through the bed reservation system based on WeChat, patients could make bed reservations at home rather than going to the hospital to make a bed reservation. Through WeChat messages, patients could receive timely notifications concerning whether their application was approved or rejected. The bed reservation system based on WeChat was easy to operate and not limited by space or time. During the COVID-19 pandemic, bed reservations through WeChat reduced the risk of infection caused by patients’ trips to and from community hospitals. Second, patients could make personalised bed reservations. They could book their preferred type of bed according to their own needs, which improved the patient experience and increased patient satisfaction. Third, the clinical department oversaw bed reservations, including approvals. Oncology patients undergo comprehensive treatment cycles, and extending the first doctor’s responsibility system to inpatient bed reservations can enhance patient trust and treatment adherence. The department’s medical staff could predetermine and verify beds, facilitating comprehensive health management and fostering communication among doctors, patients, and nurses, especially in unique circumstances. Finally, nurses did not need to manually register bed reservations or notify patients via phone or message for admission. This eliminated manual errors and reduced the nursing workload. The head nurse could perform dynamic and flexible scheduling and allocate human resources according to the total number of patients in the department, their condition, admission status, nursing workload, etc. This optimisation not only saved resources but also improved the average hours of nursing care per inpatient per day, enhancing the quality of care and ensuring nursing safety.

## Limitations

The shortcomings of this study are as follows. First, data from only 11 months before and after the implementation of bed reservations were collected and compared. To fully understand and explore the influence of bed reservations on departments’ operational efficiency and management, studies need to be conducted over longer periods. Second, bed usage information—which could only be checked by nurses who approve beds on an office computer at the nurses’ station in the department—could not be obtained in real time, which was coupled with the lack of intelligent real time information notifications. Particularly during rest days, relying on the nurses on duty to monitor the beds in the wards is imperative. Third, WeChat’s bed reservation information management platform is not integrated with the internal HIS. Thus, using our department’s WeChat public account’s bed reservation system, patients could only reserve beds and wait for nurses’ admission approval; they could not obtain bed information from the internal HIS. The process of bed reservation approval relied on manual operation by nurses and could not be carried out automatically in real time, even if the number of hospital beds was sufficient. Thus, to some degree, the patients’ bed waiting time was slightly prolonged.

## Conclusions

The main contributions of this study are as follows. First, by using the inpatient bed reservation system in the department’s official WeChat account, patients do not have to visit the hospital personally for appointment registration procedures, reducing the risk of infection in hospitals during the pandemic. In this way, patients can also promptly receive notifications concerning their bed application approval. Second, inpatient bed reservations using the WeChat account instead of manual registration and telephone notice reduced the nursing workload and the probability of errors or omissions caused by manual registration. Third, because of the right of the department’s medical staff to anticipate demand and review beds, it is convenient for the holistic process and full-cycle health management of cancer patients and for precommunication between doctors, nurses, and patients in exceptional cases. Although this study demonstrated the contribution of the reservation system to improving the department’s operational efficiency and nursing quality and optimising nursing workforce deployment, it did not investigate the patients’ and administrators’ experiences with the reservation system or its effect on improving patients’ quality of life and alleviated fatigue due to shorter hospital visits. Subsequent studies should focus on these topics. Furthermore, we will conduct a study based on semistructured interviews with patients and administrators in the future.

## Data Availability

The datasets used and/or analysed during the current study are available from the corresponding author upon reasonable request.

## Abbreviations

HIS: Hospital Information System
MNC: Missed Nursing Care
PRC: People’s Republic of China

## Glossary

Normalised prevention and control: This term was proposed during the low-risk period when the COVID-19 pandemic was being controlled. People gradually returned to their normal lives and work but needed to take protective measures, including personal protection (regular handwashing, wearing masks, etc.) and reducing gatherings. According to the requirements of the Chinese government’s prevention and control approach, regular nucleic acid testing was required. Information Management of Full-Cycle Inpatient Bed Reservation for Cancer Patients Under the Normalised Prevention and Control of the COVID-19 Pandemic.
